# Can Native T1 Mapping Differentiate between Healthy and Diffuse Diseased Myocardium in Clinical Routine Cardiac MR Imaging?

**DOI:** 10.1371/journal.pone.0155591

**Published:** 2016-05-24

**Authors:** Juliane Goebel, Ingmar Seifert, Felix Nensa, Haemi P. Schemuth, Stefan Maderwald, Harald H. Quick, Thomas Schlosser, Christoph Jensen, Oliver Bruder, Kai Nassenstein

**Affiliations:** 1 Department of Diagnostic and Interventional Radiology and Neuroradiology, University Hospital Essen, Essen, Germany; 2 Clinic of Cardiology and Angiology, Elisabeth Hospital, Essen, Germany; 3 Erwin L. Hahn Institute for Magnetic Resonance Imaging, University of Duisburg-Essen, Essen, Germany; 4 High Field and Hybrid MR Imaging, University Hospital Essen, Essen, Germany; University Hospital of Würzburg, GERMANY

## Abstract

**Objectives:**

T1 mapping allows quantitative myocardial assessment, but its value in clinical routine remains unclear. We investigated, whether the average native myocardial T1 value can be used as a diagnostic classifier between healthy and diffuse diseased myocardium.

**Methods:**

Native T1 mapping was performed in 54 persons with healthy hearts and in 150 patients with diffuse myocardial pathologies (coronary artery disease (CAD): n = 76, acute myocarditis: n = 19, convalescent myocarditis: n = 26, hypertrophic cardiomyopathy (HCM): n = 12, dilated cardiomyopathy (DCM): n = 17) at 1.5 Tesla in a mid-ventricular short axis slice using a modified Look-Locker inversion recovery (MOLLI) sequence. The average native myocardial T1 value was measured using dedicated software for each patient. The mean as well as the range of the observed average T1 values were calculated for each group, and compared using t-test. The ability of T1 mapping to differentiate between healthy and diffuse diseased myocardium was assessed using receiver operating characteristic analysis (ROC).

**Results:**

The mean T1 value of the group “healthy hearts” (955±34ms) differed significantly from that of the groups DCM (992±37ms, p<0.001), HCM (980±44ms, p = 0.035), and acute myocarditis (974±36ms, p = 0.044). No significant difference was observed between the groups “healthy hearts” and CAD (951±37ms, p = 0.453) or convalescent myocarditis (965±40ms, p = 0.240). The average native T1 value varied considerably within all groups (range: healthy hearts, 838-1018ms; DCM, 882-1034ms; HCM, 897-1043ms; acute myocarditis, 925-1025ms; CAD, 867-1082ms; convalescent myocarditis, 890-1071ms) and overlapped broadly between all groups. ROC analysis showed, that the average native T1 value does not allow for differentiating between healthy and diffuse diseased myocardium, except for the subgroup of DCM.

**Conclusions:**

The average native T1 value in cardiac MR imaging does not allow differentiating between healthy and diffusely diseased myocardium in individual cases.

## Introduction

Due to recent technical developments in cardiac magnetic resonance imaging (CMR), quantitative assessment of the myocardium has become feasible and T1, T2, and T2* times can be measured to quantify tissue properties. Different CMR techniques have been described for quantitative in vivo T1 measurement, which rely either on inversion recovery, saturation recovery, or a combination of both techniques. The sequences employed are called modified Look-Locker inversion recovery (MOLLI), shortened modified Look-Locker inversion recovery (ShMOLLI), saturation recovery single-shot acquisition (SASHA), and saturation pulse prepared heart-rate-independent inversion recovery (SAPPHIRE) [1–4]. The native T1 relaxation time is considered to be a genuine tissue property [5]. It is altered by changes of both extracellular space/ interstitium and cardiomyocytes and may be useful not only to detect focal, but also diffuse cardiac pathologies [5,6]. In general, native T1 values are elevated when the extracellular space is increased, e.g. in edema, diffuse fibrosis or focal scarring, or in interstitial amyloid deposition [4,7,8], whereas they are decreased in iron accumulation and intracellular fat deposition like in Anderson-Fabry disease [4,9]. Accordingly changes in the native T1 value have been described in various cardiac diseases, and to some extent the reported mean T1 values allowed for a differentiation between distinct patient groups [10–12]. However, it remains unclear whether the average native myocardial T1 value is a reasonable discriminator between healthy and diffuse diseased myocardium (e.g. myocarditis, HCM, DCM) in individual cases. Hence, the present study investigated whether the individual average native myocardial T1 value allows for differentiation between healthy and diffuse diseased myocardium in the clinical routine.

## Materials and Methods

### Patients

The retrospective analysis of the data was approved by the local ethic committee of the University Hospital Essen. Only data was analyzed and all patient records/ information was anonymized and de-identified prior to analysis. Between February and June 2015, 401 unselected consecutive subjects referred for CMR were primarily included in this study. Consecutively, 42 of these subjects had to be excluded due to T1 mapping artifacts caused by breathing, off-resonance, susceptibility, and ventricular motion [13]. Additionally, all subjects who did not undoubtedly fall into one of the following diagnostic categories: no cardiac disease/ healthy heart, known coronary artery disease (CAD) without myocardial infarction in the T1 mapping plane, acute or convalescent myocarditis, hypertrophic cardiomyopathy (HCM), and dilated cardiomyopathy (DCM), or who suffered from a combination of these pathologies were excluded (n = 155). Therefore a total of 204 subjects was finally included (70 female, 134 male, mean age 54.7 ± 15.5 years (range 18–87 years), mean weight 82 ± 15.9 kg (range 42–134 kg), mean body mass index 26.3 ± 4.0 kg/m^2^ (range 17.0–41.3 kg/m^2^)). Assignment to the above mentioned diagnoses was performed by two experienced cardiologists in consensus using all available clinical information (medical history, ECG, laboratory tests, coronary angiography, cardiac computed tomography) as well as the CMR results (except of T1 mapping). Subjects, who were referred for CMR as part of a preventive check-up and who did neither show any pathology in CMR nor any clinical cardiac abnormality, were defined as having a “healthy heart”. Diagnosis of myocarditis, DCM, HCM, and CAD was made in accordance to established diagnostic criteria [14–16]. Patients with myocarditis were classified as acute or convalescent depending on the presence of clinical symptoms, edema, pericardial effusion, and serological marker abnormalities [12,16,17]. Accordingly, 54 persons (26.5%) were classified as having a healthy heart, 76 patients (37.7%) were classified as suffering from CAD, 19 patients (9.3%) as suffering from acute myocarditis, 26 patients (12.7%) as suffering from convalescent myocarditis, 12 patients (5.9%) as suffering from HCM, and 17 patients (8.3%) as suffering from DCM.

### CMR imaging

All MR scans were performed on a 1.5-Tesla MRI system (Magnetom Avanto, Siemens Healthcare GmbH, Erlangen, Germany). For cine imaging steady-state free precession (SSFP) images were acquired in standard long and short axis views (TR 3.2 ms, TE 1.3 ms, matrix 192 x 174, field of view depended on the anatomy and ranged from 349 x 284 mm^2^ to 420 x 380 mm^2^, flip angle 80°, bandwidth 930 Hz/pixel, GRAPPA R = 2, 24 reference lines, slice thickness 6 mm). Native T1 mapping was performed in end-diastole using a modified Look-Locker inversion recovery (MOLLI) investigational sequence (Works-in-progress, Siemens Healthcare GmbH, Erlangen, Germany) in a single mid-ventricular short axis slice, as previously described (TR 2.63 ms, TE 1.12 ms, 8 different TIs ranging from 120 to 4103 ms, matrix 256 x 144, field of view 360 x 306 mm^2^, flip angle 35°, bandwidth 1085 Hz/pixel, GRAPPA R = 2, 24 reference lines, slice thickness 8 mm) [4,5,12,18,19]. The modified MOLLI sequence implementation in this study (5(3)3) comprised one inversion pulse with T1 sampling performed over 5 acquisition heartbeats, followed by 3 recovery heartbeats, and a second inversion pulse followed by 3 acquisition heartbeats. This modified MOLLI sequence is faster than the original MOLLI configuration (3, 3, and 5 acquisition heartbeats, followed by T1 recovery periods of 3 heartbeats each), and, more important, heart rate dependency is significantly reduced [4,19]. Ten minutes after intravenous administration of 0.15 ml gadoteric acid (Dotarem, Guerbet, Villepinte, France) per kg body weight a T1 weighted Inversion Recovery Fast Low Angle Shot (IR-FLASH) sequence was acquired in standard long and short axis views (TR 3.1 ms, TE 2.33 ms, TI (individually adjusted) ranging from 270 to 330 ms, matrix 256 x 116, field of view 360 x 306 mm^2^, flip angle 30°, bandwidth 300 Hz/pixel, GRAPPA R = 2, 24 reference lines, slice thickness 6 mm) to assess late gadolinium enhancement (LGE).

### Image analysis

**Fig 1:**
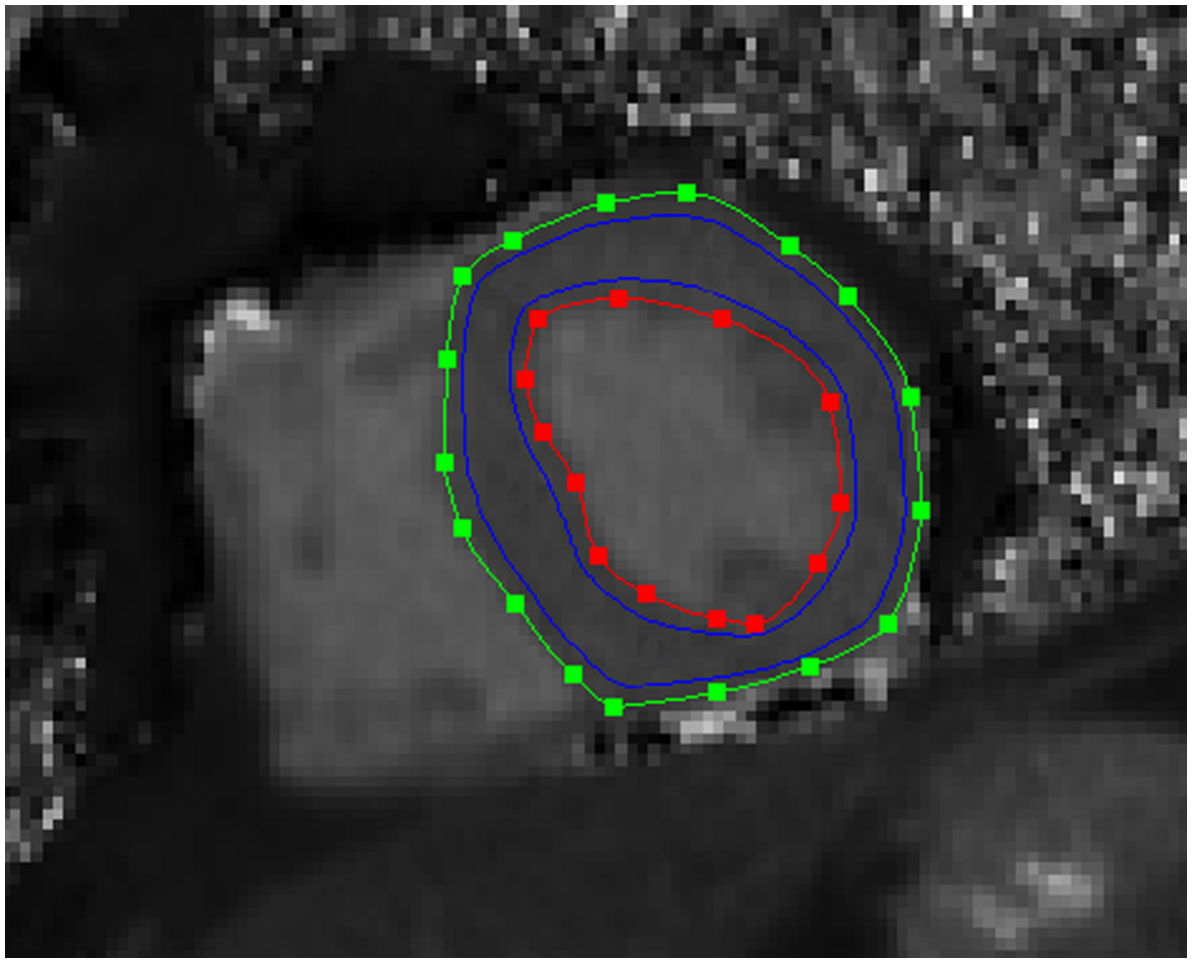
Mid-ventricular short axis native T1 map. Endocardial (red line) and epicardial (green line) contours, as well as the region-of-interest for the calculation of the average myocardial T1 value (area between the blue lines) can be seen.

Two cardiologists (>5 years and >10 years of CMR experience) and one radiologist (>10 years of CMR experience) evaluated all CMR studies, except for the T1 mapping, in consensus. Besides qualitative CMR reading, the maximal end-diastolic thickness of the interventricular septum was measured and left ventricular (LV) volumetric quantification was performed using the Argus software (Siemens Healthcare GmbH, Erlangen, Germany), as previously described [20]. Additionally, a second radiologist (>5 years of CMR experience) measured the average myocardial T1 value in the acquired mid-ventricular short axis slice using the freely available software Segment version 1.9 R4040 (http://segment.heiberg.se), while after manual definition of the endocardial and epicardial contours, 40% of the resulting myocardial area was excluded to minimize partial volume artifacts (20% at the inner endocardial rim and 20% at the outer epicardial rim; Fig 1), as previously described [5].

### Statistical analysis

Statistical analysis was done using MedCalc (version 15.6, MedCalc Software, Belgium). A D’Agostino-Pearson test was used to test for normal distribution. Normally distributed data are given as mean ± standard deviation (SD). Categorical data are given as absolute numbers or frequencies. Testing for differences of average T1 values and volumetric data between the different groups (healthy individuals and the different patient groups with diffuse heart disease) was performed using unpaired t-test. Receiver operating characteristic (ROC) analysis was used to test, whether the average native T1 value allows distinguishing between healthy and diffused diseased myocardium. Statistical significance was defined as p < 0.05.

## Results

Demographic features as well as detailed analysis of LV structural and functional parameters (end-diastolic volume, end-systolic volume, stroke volume, ejection fraction, end-diastolic interventricular septum thickness) are given for the group of subjects with healthy hearts and all assessed cardiac pathologies in Table 1. Statistical analysis showed a significantly higher LV end-diastolic volume, end-systolic volume, and stroke volume and a significantly lower ejection fraction in patients with DCM compared to subjects with healthy hearts (Table 2). In HCM a significantly higher end-diastolic septum thickness was seen compared to subjects with healthy hearts (p < 0.001). Patients with acute myocarditis showed a significantly lower ejection fraction compared to healthy hearts (p = 0.030). And in patients with convalescent myocarditis a significantly higher end-diastolic volume and end-systolic volume and a significantly lower ejection fraction were seen compared to healthy hearts (Table 2). In CAD a significantly lower stroke volume and ejection fraction and a significantly higher end-diastolic septum thickness was found compared to subjects with healthy hearts (Table 2).

**Table 1:** Demographic and CMR data.

		HH	CAD	DCM	HCM	AM	CM
*n*		54	76	17	12	19	26
*Demographics*							
Age [years]	mean±SD (range)	48±10.5 (18–63)	66±10.7 (32–84)	58±16.7 (25–87)	53±12.6 (22–76)	43±16.1 (19–73)	44±15.7 (20–78)
Female/ Male	n/n	23/31	18/58	8/9	4/8	9/10	8/18
*Mean native T1 value [ms]*							
	mean±SD (range)	955±33.5 (838–1018)	951±37.3 (867–1082)	992±37.3 (882–1034)	980±43.6 (897–1043)	974±35.9 (925–1025)	965±39.5 (890–1071)
*LV quantification*							
EDV [ml]	mean±SD (range)	148±30.5 (82–204)	145±52.7 (87–372)	240±53.1 (181–345)	141±74.0 (74–338)	179±95.6 (85–526)	189±79.1 (71–447)
ESV [ml]	mean±SD (range)	57±13.5 (28–82)	71±52.0 (30–310)	169±54.5 (103–295)	63±51.3 (17–199)	97±99.7 (32–478)	92±66.6 (15–358)
SV [ml]	mean±SD (range)	91±19.6 (54–137)	74±21.8 (28–108)	71±27.5 (29–124)	77±37.9 (28–145)	82±27.6 (36–133)	97±29.1 (56–189)
EF [%]	mean±SD (range)	62±3.6 (56–70)	59±9.5 (17–73)	30±11.1 (13–44)	63±12.3 (41–79)	54±15.3 (10–74)	55±11.6 (20–78)
IVS [mm]	mean±SD (range)	10±1.2 (7–12)	11±1.4 (9–15)	10±1.9 (7–15)	20±3.3 (16–26)	10±1.9 (8–14)	10±1.5 (8–14)
*LGE* within the							
entire myocardium	n (%)	0 (0.0)	17 (22.4)	6 (35.3)	9 (75.0)	17 (89.5)	20 (76.9)
T1 mapping slice	n (%)	0 (0.0)	0 (0.0)	4 (23.5)	8 (66.7)	7 (36.8)	10 (38.5)

Abbreviations: EDV, end-diastolic volume; ESV, end-systolic volume; SV, stroke volume; EF, ejection fraction; IVS, interventricular septum thickness; LGE, late gadolinium enhancement

Demographic and CMR data of the subjects with healthy hearts (HH), patients with coronary artery disease (CAD), dilated cardiomyopathy (DCM), hypertrophic cardiomyopathy (HCM), acute myocarditis (AM), and convalescent myocarditis (CM).

**Table 2:** Comparison of native T1 values and volumetric data between subjects with healthy hearts and patients with distinct cardiac pathologies.

*P values*	HH vs. CAD	HH vs. DCM	HH vs. HCM	HH vs. AM	HH vs. CM
*Mean native T1 value*	0.453	<0.001*	0.035*	0.044*	0.240
*LV quantification*					
EDV	0.739	<0.001*	0.741	0.189	0.022*
ESV	0.128	<0.001*	0.718	0.100	0.017*
SV	0.002*	0.007*	0.279	0.204	0.366
EF	0.007*	<0.001*	0.783	0.030*	0.007*
IVS	<0.001*	0.982	<0.001*	0.296	0.189

Abbreviations: EDV, end-diastolic volume; ESV, end-systolic volume; EF, ejection fraction; IVS, interventricular septum thickness; LGE, late gadolinium enhancement; vs., versus;

*, significant

Mean value comparison of native T1 values and volumetric data (results given as p values) between the groups healthy hearts (HH), coronary artery disease (CAD), dilated cardiomyopathy (DCM), hypertrophic cardiomyopathy (HCM), acute myocarditis (AM), and convalescent myocarditis (CM).

**Fig 2:**
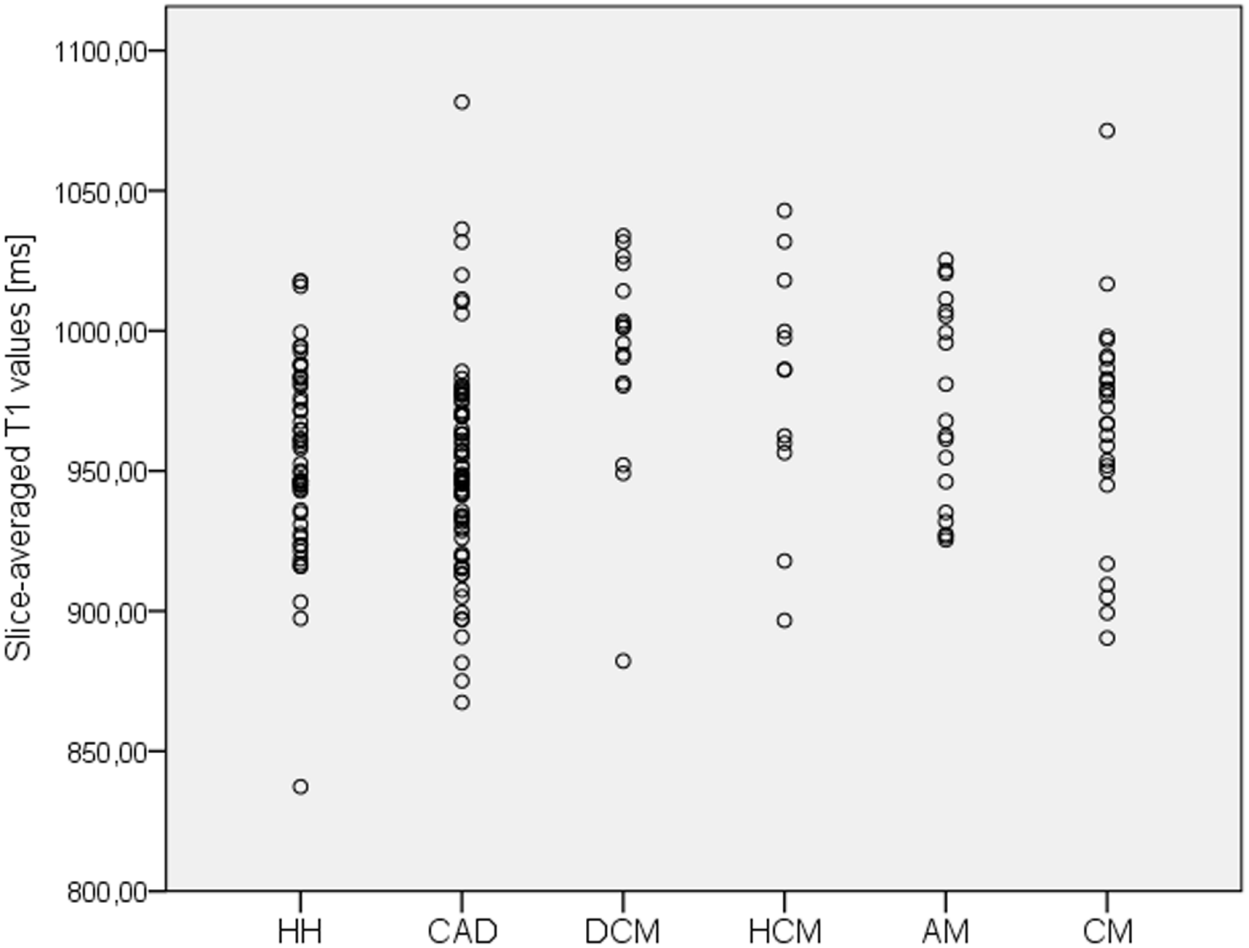
Native myocardial T1 values in healthy hearts and in several cardiac pathologies. Average native myocardial T1 values (given as dot plots) observed in the group healthy hearts (HH), coronary artery disease (CAD), dilated cardiomyopathy (DCM), hypertrophic cardiomyopathy (HCM), acute myocarditis (AM), and convalescent myocarditis (CM).

The average native myocardial T1 value ranged in the group of subjects with healthy hearts between 838 and 1018 ms (Fig 2), and its mean was 955 ± 33.5 ms. The average native myocardial T1 value ranged in the joint analysis of all patients with diffuse diseased myocardium between 867 and 1082 ms (Fig 2), and its mean was 963 ± 40.3 ms. Statistical analysis showed no significant difference between the group of subjects with healthy hearts and the group of patients with diffuse diseased myocardium (p = 0.206).

Subgroup analysis revealed a significant difference in the mean of the average native T1 value between the group of subjects with healthy hearts and the group of patients suffering from DCM (p < 0.001), the group of patients suffering from HCM (p = 0.035), and the group of patients suffering from acute myocarditis (p = 0.044). No significant differences were observed between the group healthy hearts and the group convalescent myocarditis (p = 0.240) or between the group healthy hearts and the group CAD (p = 0.453). Overall, data analysis revealed six outliners. But as outliner exclusion did not lead to a relevant change in statistical results (test for mean average native T1 value differences between the group of subjects with healthy hearts and the groups of: DCM p < 0.001, HCM p = 0.037, acute myocarditis p = 0.053, convalescent myocarditis p = 0.379, and CAD p = 0.082), these outliners were not excluded to not restrict the general validity of the study.

**Fig 3:**
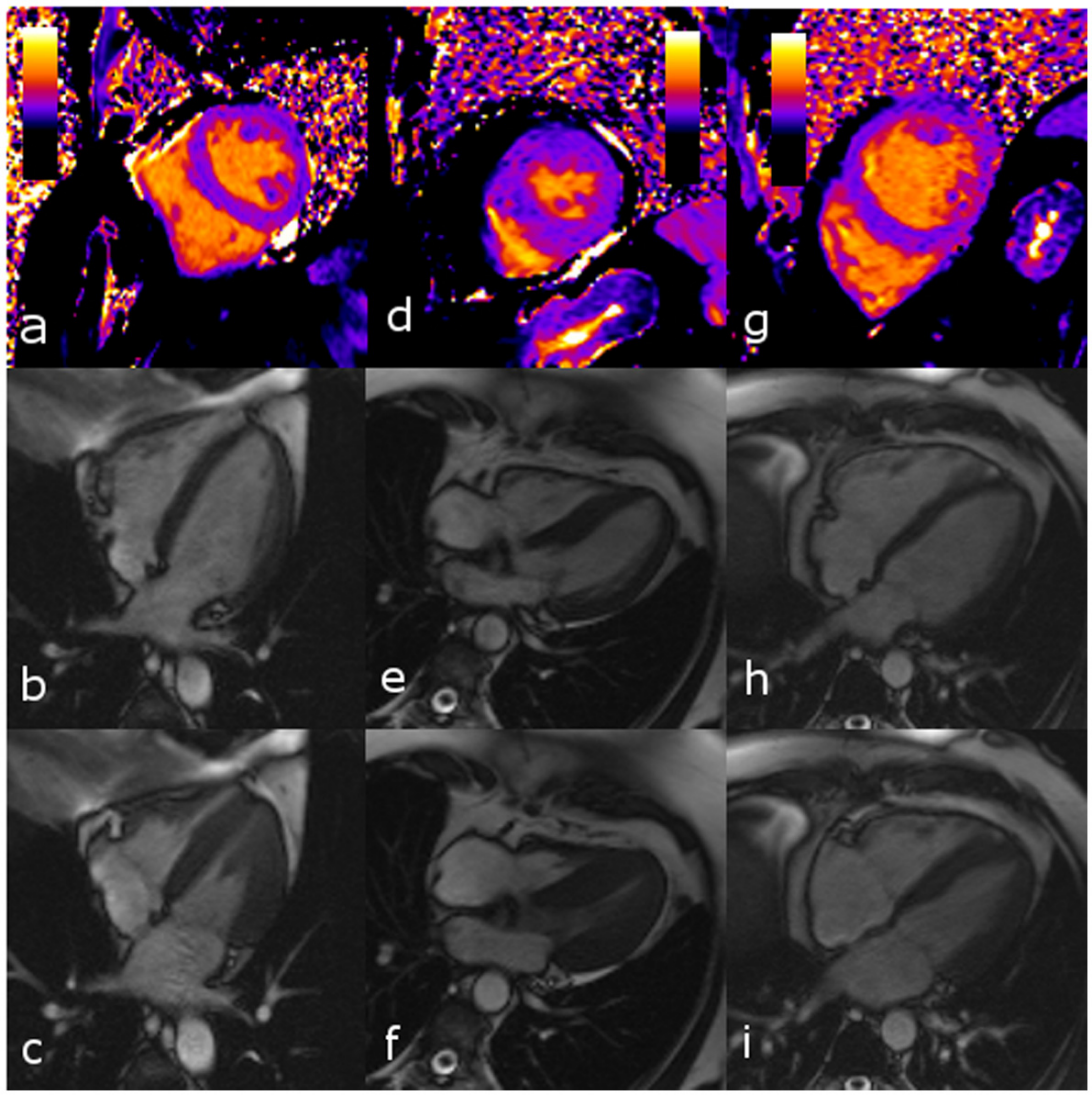
Native T1 maps (a, d, g) and cine 4-chamber images in end-diastole (b, e, h) and in end-systole (c, f, i) in a subject with a healthy heart (a-c), a patient with hypertrophic cardiomyopathy (d-e), and a patient with acute myocarditis (f-i) presenting with nearly identical slice-averaged T1 values. Native slice-averaged T1 value (T1), end-diastolic volume (EDV), end-systolic volume (ESV), ejection fraction (EF), and thickness of the interventricular septum (IVS) were as following: subject with healthy heart: T1, 968 ms; EDV, 158 ml; ESV, 58 ml; EF, 63%; IVS, 10 mm; patient with hypertrophic cardiomyopathy: T1, 963 ms; EDV, 74 ml; ESV, 17 ml; EF, 77%; IVS, 21 mm; patient with acute myocarditis: T1, 963 ms; EDV, 236 ml; ESV, 103 ml; EF, 56%; IVS, 11 mm. (The T1 scale extends from 0 ms (black) to 2000 ms (yellow).)

**Fig 4:**
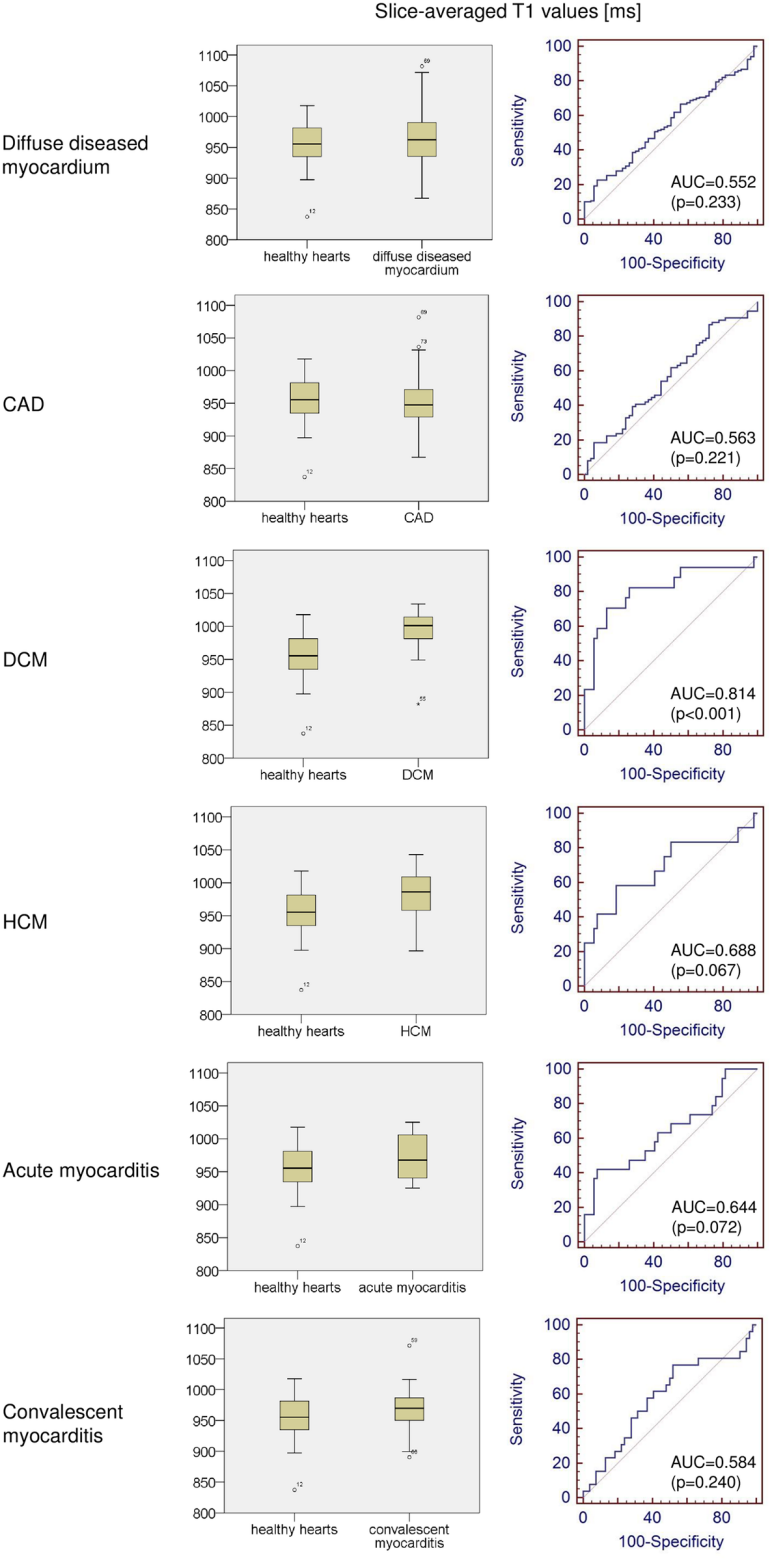
Comparison of native T1 values between healthy hearts and several myocardial pathologies. Boxplots and receiver operating characteristic curves, comparing slice-averaged native T1 values between subjects with healthy hearts and all patients with diffuse diseased myocardium, followed by individual comparisons between subjects with healthy hearts and patients with distinct cardiac diseases, can be seen. CAD, coronary artery disease; HCM, hypertrophic cardiomyopathy; DCM, dilated cardiomyopathy; AUC, area under the curve.

A significant inter-individual variance of the average native T1 values was observed in all analyzed groups (Fig 2; Table 1), as well as a substantial overlap between the observed groups (Figs 2–4). Trying to differentiate healthy hearts from diseased hearts using the average native myocardial T1 value as discriminator, ROC analysis resulted in an area under the curve (AUC) of 0.552 (p = 0.233) for joint analysis of all diffuse diseased hearts, in an AUC of 0.563 (p = 0.221) for CAD, in an AUC of 0.644 (p = 0.072) for acute myocarditis, in an AUC of 0.584 (p = 0.240) for convalescent myocarditis, in an AUC of 0.688 (p = 0.067) for HCM, and in an AUC of 0.814 (p < 0.001) for DCM (Fig 4). Thus, only for DCM the AUC was significantly different from 0.5. Demanding a specificity of 87% for the diagnosis of DCM, the slice-averaged native T1 cut-off value was >988 ms and the sensitivity was only 70.6%.

## Discussion

This cardiac MR imaging study investigated, whether the average native myocardial T1 relaxation time allows distinguishing between healthy and diffuse diseased myocardium. Our study shows that the native myocardial T1 relaxation time allows distinguishing between certain groups of patients, but that this parameter fails to distinguish between healthy and diffuse diseased myocardium in individual subjects.

Although the native T1 relaxation time is a genuine tissue parameter at a given field strength, it is well known, that T1 values measured in clinical routine are affected not only by the type of sequence used but although by sequence parameters as well as by the MR system itself.

Therefore, comparison of our data with previous studies is only possible to a limited degree. Messroghli et al., who used a MOLLI sequence at 1.5 T on a Philips Gyroscan Intera MR system, reported a mean native T1 value of 977 ± 63 ms for subjects with healthy hearts [13]. Karamitsos et al., who used a shortened MOLLI (ShMOLLI) sequence on an identical MR system as we used in this study, reported a mean native T1 values of 958 ms in subjects with healthy hearts [11], which is very comparable to our mean value of 955 ± 33.5 ms. Sado et al., who used an identical ShMOLLI sequence and an identical MR system like Karamitsos et al., reported recently a native myocardial T1 value of 968 ms for healthy volunteers, whereas Ferreira et al reported a native T1 value of 944 ms for healthy volunteers when using an identical sequence and MR system [7,9]. Overall, it could be stated that our mean native myocardial T1 value of 955 ± 33.5 ms for healthy individuals is in line with previous reports despite the fact, that small differences exist due to different sequences and MR systems used. Comparably to our findings, Messroghli et al. and Sado et al. found a relatively wide range of the average native myocardial T1 value in healthy volunteers (Messroghli et al.: 887 ms to 1105 ms, SD of the mean: 63 ms; Sado et al.: 900 ms to 1060 ms, SD of the mean 32 ms; present study: 838 ms to 1018 ms, SD of the mean 33.5 ms) [9,13]. In contrast, Karamitsos et al. and Ferreira et al. reported with 17 ms respectively 20 ms a very small standard deviation of the mean myocardial T1 value in healthy subjects, which suggests a small range of the average myocardial T1 value in their study cohorts [7,11]. However, since Ferreira et al. provided only the standard deviation and not the range of their observed values it remains unclear whether the authors observed really a small intra-individual variance of the native myocardial T1 value in their studies, or not. In any case, the average native myocardial T1 value shows a certain variance between healthy individuals in these studies.

In agreement to previously published studies, our results show that the assessment of the average native myocardial T1 relaxation time can be used to differentiate between certain patient groups since we found, that the mean native myocardial T1 value of the DCM, HCM, and acute myocarditis group differed significantly from that of the group of healthy individuals. The higher mean native myocardial T1 value in the DCM and HCM group can be explained by an increased interstitial space in DCM and HCM due to diffuse myocardial fibroses [8]. Concordant to our results Dass et al. and Puntmann et al. reported significantly higher T1 values in HCM and DCM compared to healthy individuals, even though a direct comparison of their values to ours is not possible since Dass et al. and Puntmann et al. used 3 T MR systems [8,21]. In accordance to previous reports, we found a slightly higher mean native myocardial T1 value in the group of patients suffering from acute myocarditis compared to the group of healthy individuals, which can be explained by a diffuse myocardial edema in acute myocarditis [12]. In accordance to a previous study from Hinojar et al. we found a trend towards a slightly higher mean native myocardial T1 value in our group of patients with convalescent myocarditis compared to our group of healthy individuals, however this trend did not reach statistical significance in the present study [12]. The most likely explanation for this is that the extent of residual myocardial inflammation and post-inflammatory fibroses must have been lower in our study population compared to that in the study from Hinojar et al.

In addition to DCM, HCM, and myocarditis, we assessed the native myocardial T1 values in patients with CAD, whereas we enrolled only patients without myocardial infarction in the mid-ventricular short axis slice used for T1 mapping. Similar to the above mentioned pathologies CAD leads to a diffuse myocardial injury, since it is well known, that coronary micro-embolization frequently occurs in CAD, which results in multiple myocardial micro-infarcts mainly in the mid-myocardial layers [22]. From a pathophysiological point of view an increased native myocardial T1 value can be expected due to focal myocardial edema in case of acute micro-embolization or due to multiple focal areas of fibrosis after “healed” coronary micro-embolization. However, our study shows that no difference exists in the mean native myocardial T1 relaxation time between the groups of patients suffering from CAD and healthy individuals, which shows that the structural changes in non-infarcted myocardium in CAD are obviously too small to alter significantly the native myocardial T1 relaxation time.

Although the calculation of the average native myocardial T1 relaxation time may be helpful to distinguish between certain groups of patients with different cardiac pathologies, our study shows that overall the native myocardial T1 value fails to discriminate between healthy and diffuse diseased myocardium in individual cases. Our ROC analysis showed that only in case of DCM the native myocardial T1 relaxation time allows distinguishing healthy and diffuse diseased myocardium, and even in this case the sensitivity is comparably low. Reflecting individual native myocardial T1 values instead of analyzing mean values in patient groups shows the reason for the poor performance of the native myocardial T1 value as discriminator between healthy and diffuse diseased myocardium in individual cases: the substantial inter-individual variance of the native myocardial T1 value both in healthy individuals as well as in diffuse myocardial pathologies. Unfortunately, the majority of studies concerning native T1 mapping in diffuse myocardial pathologies focused on the analysis of differences in the mean value between different groups of patients and healthy volunteers, despite the fact that the differentiation between healthy and diffuse diseased myocardium on individual patient level is from greater clinical interest.

However, it must be noted, that some studies reported, that native T1 mapping allows discrimination between healthy and diffuse diseases myocardium in individual cases. Hinojar et al. reported recently, that the native myocardial T1 value allows distinguishing between acute myocarditis and healthy hearts with a sensitivity of 96% and specificity of 100% [12]. While these values are very high and suggest excellent sensitivity and specificity, they are diametrically opposed to our findings. However, further studies reported, that the native T1 value allows discriminating between healthy and diffuse diseased myocardium in individual cases in further pathologies: While Ferreira et al. reported, that the native T1 value allows discrimination between healthy myocardium and Takotsubo cardiomyopathy with a sensitivity and specificity of 92% (AUC = 0.94) when a threshold of 990 ms is used in combination with ShMOLLI based T1 mapping on a 1.5 T system [7], Karamitsos et al. reported a high diagnostic accuracy for the discrimination between patients with definite cardiac amyloidosis and healthy myocardium [11]. Therefore, the question arises what the reason for the divergence between T1 mapping results in the cited studies and in the present study might be. To our firm belief the most plausible explanation for this discrepancy is patient selection. When considering only patients with a maximum extent of disease, it might be possible that the native T1 value allows discriminating between healthy and diffuse diseased myocardium with an excellent diagnostic accuracy. Exemplary analysis of the study population of Hinojar et al., who found a very high diagnostic accuracy, supports this assumption: While the healthy individuals had a mean left ventricular mass index of 50 g/m², the patients with acute myocarditis had a mass index of 70 g/m² in their study, which can be explained only by a massive myocardial edema. Concordantly, Hinojar et al. stated that “in the acute myocarditis group, patients exhibited visually detectable increases in T2 signal”. Moreover they reported a high frequency of late gadolinium enhancement in their study population. Thus, it is highly likely, that only patients with severe disease had been included in their study, while patients with moderate extent of disease had been left unconsidered, which is opposite to our approach which might, in this regard, better reflect clinical reality.

The present study has some limitations. First and foremost, like in previous studies we performed T1 mapping for practical reasons in regard to the overall CMR acquisition length only in one mid-ventricular short axis slice [12,18]. Therefore, the measured average native myocardial T1 value in this slice may not fully represent the entire LV myocardium. However, in pathologies, which diffusely affect the entire LV myocardium like myocarditis, HCM, or DCM this seems to be negligible. The second limitation of our study is that we excluded 20% of the inner- and outermost myocardium in order to avoid partial volume artifacts, which is in accordance with the recommendation of the European society of cardiology CMR working group [5]. Last, the number of patients in some subgroups of diffuse myocardial pathologies (e.g. HCM) is rather small.

In conclusion, our question, whether native T1 mapping in cardiac MR imaging can differentiate between healthy and diffuse diseased myocardium, must be answered with “yes” and “no”, since the native myocardial T1 relaxation time allows discriminating between groups of patients with certain diffuse myocardial pathologies and a group of healthy individuals, but does not allow differencing between healthy and diffuse diseased myocardium in individual subjects.

## Supporting Information

S1 Table Demographic and magnetic resonance imaging raw data. (XLSX)

## Abbreviations

AUC: area under the curve
CAD: coronary artery disease
CMR: cardiac magnetic resonance imaging
DCM: dilated cardiomyopathy
HCM: hypertrophic cardiomyopathy
LGE: late gadolinium enhancement
LV: left ventricular
SD: standard deviation
